# Cholecystostomy as Bridge to Surgery and as Definitive Treatment or Acute Cholecystectomy in Patients with Acute Cholecystitis

**DOI:** 10.1155/2016/3672416

**Published:** 2015-12-29

**Authors:** Agnieszka Popowicz, Lars Lundell, Peter Gerber, Ulf Gustafsson, Emil Pieniowski, Helen Sinabulya, Krister Sjödahl, Andrianos Tsekrekos, Gabriel Sandblom

**Affiliations:** ^1^Department of Surgical Gastroenterology, GastroCentrum, Karolinska University Hospital, Huddinge CLINTEC Karolinska Institutet, 141 86 Stockholm, Sweden; ^2^Department of Surgery, S:t Göran Hospital, S:t Göransplan 1, 112 81 Stockholm, Sweden; ^3^Department of Surgery, Danderyd Hospital, Mörbygårdsvägen, 182 88 Stockholm, Sweden; ^4^Department of Surgery, Söder Hospital, Sjukhusbacken 10, 118 83 Stockholm, Sweden; ^5^Department of Surgery, Södertälje Hospital, Rosenborgsgatan 6-10, 152 86 Södertälje, Sweden; ^6^Department of Surgery, Norrtälje Hospital, TioHundra AB, P.O. Box 905, 761 29 Norrtälje, Sweden

## Abstract

*Purpose.* Percutaneous cholecystostomy (PC) has increasingly been used as bridge to surgery as well as sole treatment for patients with acute cholecystitis (AC). The aim of the study was to assess the outcome after PC compared to acute cholecystectomy in patients with AC.* Methods.* A review of medical records was performed on all patients residing in Stockholm County treated for AC in the years 2003 and 2008.* Results.* In 2003 and 2008 altogether 799 and 833 patients were admitted for AC. The number of patients treated with PC was 21/799 (2.6%) in 2003 and 50/833 (6.0%) in 2008. The complication rate (Clavien-Dindo ≥ 2) was 4/71 (5.6%) after PC and 135/736 (18.3%) after acute cholecystectomy. Mean (standard deviation) hospital stay was 11.4 (10.5) days for patients treated with PC and 5.1 (4.3) days for patients undergoing acute cholecystectomy. After adjusting for age, gender, Charlson comorbidity index, and degree of cholecystitis, the hospital stay was significantly longer for patients treated with PC than for those undergoing acute cholecystectomy (*P* < 0.001) but the risk for intervention-related complications was found to be significantly lower (*P* = 0.001) in the PC group.* Conclusion.* PC can be performed with few serious complications, albeit with a longer hospital stay.

## 1. Introduction

Laparoscopic cholecystectomy has during the last two decades become the therapeutic strategy for acute cholecystitis (AC) [1]. Even though early cholecystectomy is the therapeutic modality of preference for AC, it can in patients with high risk for surgical interventions result in significant morbidity and mortality [2]. In the 1980s percutaneous cholecystostomy (PC) was introduced as a minimally invasive procedure for decompression and drainage of the gallbladder, in patients with AC with or without severe sepsis, who were considered to have a high risk associated with emergency surgery [3–5]. Conservative management of acute cholecystitis, with percutaneous cholecystostomy and subsequent elective cholecystectomy, following appropriate preoperative assessment and preparation, is an alternative to early cholecystectomy that has not been fully evaluated [6].

Therapeutic approaches regarding disorders of the gastrointestinal tract develop continuously. Examples are the nonsurgical management of acute diverticulitis and appendicitis [7, 8]. Similarly, given that efficient control of the inflammatory state is achieved, fragile and aged patients with high comorbidity and AC who due to current clinical state are not chosen for early cholecystectomy may respond favorably to conservative management [9, 10]. In several studies PC followed by late laparoscopic cholecystectomy has been suggested as suitable management for patients with AC considered unfit for emergency surgery [5, 11, 12].

The aim of the present study was to explore the outcome after PC in a population-based cohort consisting of all patients residing in Stockholm County in 2003 and 2008 and to assess effectiveness and safety of PC and AC in comparable patient groups. In order to survey the use of PC over time we identified two cohorts at an interval of five years.

## 2. Material and Methods

Two one-year periods, separated by four years, were investigated (2003 and 2008). All patients residing in Stockholm County who were treated for AC during the study periods were identified through a search in the National Patient Register (The National Board of Health and Welfare), using personal registration numbers for linking [13]. A review of the medical records was performed according to a standard protocol. Seven hospitals contributed data to this study. The demographic characteristics of the 799 patients admitted in 2003 and of the corresponding 850 from 2008 are given in Table 1.

The comorbidity of the patients was quantified by the Charlson comorbidity index [14]. Treatment for each patient was determined by the individual physician in charge, a decision-making process which was affected by factors such as comorbidity, day-to-day variations in case load, and asset of OR resources and facilities. The guidelines for treatment of cholecystitis changed somewhat over the years but in 2008 they were strictly in favor of early cholecystectomy. During the same time period no specific guidelines were available for the indications and use of cholecystostomy in the management of acute cholecystitis.

The insertion of the cholecystostomy tube was performed by a radiologist, under local anesthesia and using ultrasound guidance. Complications related to the cholecystostomy were registered. No registration was made regarding the time to withdraw or planned controls of the tube.

The severity of cholecystitis was assessed according to the Tokyo guidelines [15]. Those presenting with cholangitis were not included in the present series. Patients with cholecystitis alone, but treated conservatively, were scheduled for an elective laparoscopic cholecystectomy after discharge. The indication for delayed cholecystectomy, in patients having a cholecystostomy tube as a bridge to elective surgery, was finally evaluated by the individual physician in charge. The same was also true for the other patients regarding the design and content of follow-up.

Postoperative complications were defined as adverse events related to each respective intervention within 30 days. The complications were scored according to the Clavien-Dindo system [16].

If a subsequent elective cholecystectomy was performed, the PC was defined as the first intervention in a bridge to surgery approach. If no subsequent surgery was undertaken, the PC was defined as definitive treatment. Recurrence of AC was defined as a new admission due to gallstone pain or AC. If planned surgery was performed, it was counted as a censored event. The hospital stay related to a recurrent AC or subsequent surgery was not included in the estimation of hospital stay.

The follow-up of each patient and survey of the outcome were completed at December 31, 2011.

### 2.1. Statistics and Validation of Data

Differences in hospital stay were tested using multivariate regression analysis, with adjustment for age (with 70 years as cut-off), gender, degree of cholecystitis (Tokyo guidelines 1 versus 2-3), and Charlson comorbidity index (0-1 versus ≥2). Difference in the risk for postoperative complications (≥Clavien-Dindo 2) was tested in a multivariate logistic analysis using the same covariates. Data on complications abstracted from the patient records were not reliable enough for Clavien-Dindo 1, so only Clavien-Dindo 2 or higher were included in the analyses.

Time to readmission for cholecystitis was analyzed using Cox proportional hazard analysis with the same covariates. When measuring time to readmission, planned surgery and death from causes not related to the cholecystitis were considered censored events.

## 3. Results

There was a stable number of patients admitted for cholecystitis during the two study periods (2003 and 2008). The slight preponderance of females remained, as well as the number of readmissions (Table 1). Moreover, no change in mean age of the patients at the first admission was observed.

Early cholecystectomy was performed in close to half of those who had cholecystitis in 2003 (Table 1). Patients treated with cholecystostomy, as a bridge to elective surgery, were older with a dominance of females. The number of patients treated with cholecystostomy increased from 21/799 (2.6%) in 2003 to 50/833 (6.0%) in 2008. Mean hospital stay for patients treated with PC was 11.4 days. The degree of inflammation was more severe in those who had cholecystostomy as compared to those having an early cholecystectomy. The severity of cholecystitis was similarly scored in those having an early operation and those treated conservatively, as well as in those who ultimately had a delayed-elective cholecystectomy.

The hospital stay was almost doubled in those treated with cholecystostomy, but otherwise the complication rate, as scored according to Clavien-Dindo, was lower than those treated with acute surgery 2.8% versus 17.1%. The complications reported in the “bridge to surgery” group were entirely confined to the subsequent final gallbladder operation. The complications in the PC group were as follows: Clavien-Dindo 2: one urinary retention and one local infection around the cholecystostomy, Clavien-Dindo 3: one abdominal abscess, and Clavien-Dindo 5: septicemia and myocardial infarction. After adjusting for age, gender, Charlson comorbidity index, and degree of cholecystitis, the hospital stay was found to be significantly longer for patients treated with cholecystostomy compared to those undergoing early cholecystectomy (*P* < 0.001). On the other hand the risk for intervention-related complications was found to be significantly lower (*P* = 0.001) in the cholecystostomy group when adjusting for the same covariates. Hospital stay was tested with univariate and multivariate regression analysis, and complication rate was tested with univariate and multivariate logistic regression analysis, with adjustment for age, gender, Charlson comorbidity index, and degree of cholecystitis (Tables 2(a) and 2(b)).

**Figure 1:**
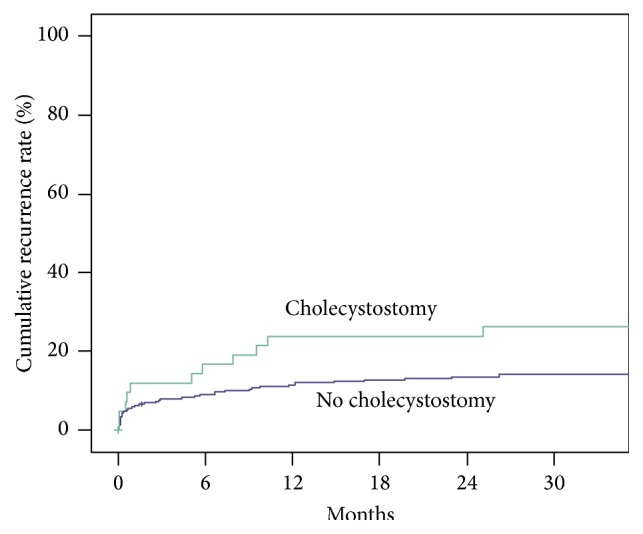
Cumulative recurrence rate after the first admission for patients treated with cholecystostomy and patients treated conservatively without intervention. The Kaplan-Meier plot was constructed by defining readmission for recurrent cholecystitis as terminal event and end of follow-up (December 31, 2011), death, or planned cholecystectomy as censored events. The difference between the groups was not statistically significant in univariate (*P* = 0.056) or multivariate (*P* = 0.051) Cox proportional hazard analysis with adjustment for age, gender, Charlson comorbidity index, and degree of cholecystitis.

The risk for readmission for new episodes of cholecystitis did not differ significantly between the cholecystostomy group and the rest of the cohort when adjusting for the same covariates. The recurrence rate of cholecystitis within one year was found to be 28% for patients treated with PC and 19% for those treated conservatively.  Figure 1 shows the cumulative recurrence rate after the first admission for patients treated with cholecystostomy and patients treated conservatively without intervention for the three-year follow-up period. The Kaplan-Meier plot was constructed by defining readmission for recurrent cholecystitis as terminal event and end of follow-up (December 31, 2011), death, or planned cholecystectomy as censored events. The difference between the groups was not statistically significant in univariate (*P* = 0.056) or multivariate (*P* = 0.051) Cox proportional hazard analysis with adjustment for age, gender, Charlson comorbidity index, and degree of cholecystitis.

## 4. Discussion

Cholecystectomy is currently the recommended treatment for AC. This procedure, however, is implemented in approximately half of the patients admitted to hospital with this diagnosis. Similar figures have also been reported from the USA [17, 18]. This indicates that in the clinical practice a nonsurgical approach is often chosen despite guidelines that recommend acute cholecystectomy. This study provides outcome data after acute surgery and PC in comparable patient groups. Even if more complications may be expected after a surgical approach, the question remains whether the complication rate can be considered acceptable. In this study adjustments are made for age, comorbidity, gender, and the severity of the cholecystitis, but there is still a risk for residual confounding.

A higher complication rate was noticed in the early surgery group compared to that reported in previous literature. The data from the present study reflect the surgical outcome as practiced in the community at large, whereas many of the previous studies presenting fewer complications have been performed at specialized units, with dedicated surgeons and staffs. The complication rate was close to the rates reported from the Swedish Register for Gallstone Surgery and ERCP [19].

It seems as there was no great change in therapeutic strategy between the years 2003 and 2008 covered by the present survey. However, during the same period a significant increase in the use of PC was noted, both as definite treatment and as a bridge to later elective cholecystectomy. As expected patients selected for treatment with PC were older, having a higher Charlson comorbidity index and a higher severity grade of the gallbladder inflammation. This may well have been the reason why PC patients had a longer hospital stay, which otherwise could not be explained by other confounding factors. PC as such was not burdened by procedure-related complications. Instead, those who subsequently had a delayed cholecystectomy were those who encountered complications. Noteworthy was that the severity of cholecystitis was similarly scored in those having an emergency surgery and PC and treated conservatively and those who ultimately had a delayed-elective cholecystectomy. This shows that there is a selection bias due to the surgeon's choice of treatment and experience, the patients comorbidity, guidelines, and asset of OR resources. We believe that these results show a reality in medical care in hospitals both on University level and smaller hospitals level. During the follow-up of patients treated with PC we did not observe an enhanced admission rate due to AC. Similarly, McGillicuddy and colleagues noted that patients treated with antibiotics alone or with PC had a low recurrence rate of AC and suggested that this therapy is sufficient for patients with high risk for general anesthesia [10]. Our data and experience differ from the results presented in a recent review of the literature on the clinical outcome of patients with AC managed with PC. In this review the authors concluded that there appeared to be a 15% 30-day mortality rate following PC, with substantial variability in complication rates between the studies [20]. With increasing use and experience of PC, this intervention can be performed with very few serious complications [5]. This was evident also from results of the present study, where an increase in the use of the procedure was seen over a six-year period. In fact the significant morbidity observed was confined to the subsequent cholecystectomy and not the PC as such.

PC can be an attractive alternative to early surgery in elderly, fragile patients particularly if the severity score of the AC is judged significant and offers an effective bridge to definitive surgery, but the critical question is the timing of that operation and which patients ultimately require cholecystectomy. The follow-up period in this study was longer than three years for all patients, which is comparatively long. During this period, we observed a limited number of readmissions for AC, illustrating that PC can offer a definite treatment for some AC patients. However, future studies should carefully explore the characteristics of patients suitable for PC alone. As a treatment modality an advantage would be if the catheter could be withdrawn as soon as possible in order to facilitate the rehabilitation of the respective patient. In this context it is important to emphasize adherence to the principle of performing fluoroscopic assessment of cystic duct patency before extraction of the PC catheter [21, 22].

A particularly relevant question is the place of PC for those AC patients who present with a “long” history, traditionally considered to be associated with a high complication rate if early cholecystectomy is performed. AC patients with a history > 7 days are traditionally managed conservatively due to an alleged increase in risk with cholecystectomy at that stage in the disease process [15]. It is quite reasonable to suggest that, in a similar situation, PC may have an important role to play by offering these patients a safer delayed gallbladder operation at a later stage.

The present study is limited by the retrospective design. Even though it is population-based, there may be local variations in the way PC is practiced and when PC is decided on. In order to draw definite conclusion regarding the safety of the treatment, randomized controlled trials should be performed. Acute cholecystectomy should be compared to PC as bridge to surgery, being the two alternatives for definite treatment. Similarly, PC should be compared to conservative management for patients with high comorbidity in prospective studies.

## 5. Conclusion

PC is a safe option to emergency surgery for patients with AC who are considered to be at high risk for surgical intervention. It can be performed with few serious complications, albeit with a longer hospital stay.

## Figures and Tables

**Table 1:** Patient characteristics. Information about management was missing or inconsistent for 284 patients.

	Conservative management, no intervention (*N* = 293)	Early surgery (*N* = 736)	Cholecystostomy as sole treatment (*N* = 61)	Cholecystostomy as bridge to surgery (*N* = 10)	Delayed surgery (*N* = 248)
Women	157 (53.6%)	403 (54.8%)	35 (57.4%)	8 (80.0%)	146 (58.9%)
Men	136 (46.4%)	333 (45.2%)	26 (42.6%)	2 (20.0%)	102 (41.1%)

Mean age, years (standard deviation)	72 (15)	52 (17)	79 (13)	64 (15)	56 (15)

Charlson comorbidity index					
0	99 (33.8%)	562 (76.4%)	8 (13.1%)	4 (40.0%)	155 (62.5%)
1	80 (27.3%)	121 (16.4%)	14 (23.0%)	3 (30.0%)	46 (18.5%)
≥2	113 (38.6%)	48 (6.5%)	39 (63.9%)	3 (30.0%)	45 (18.1%)
Data missing	1 (0.3%)	5 (0.7%)	0 (0%)	0 (0%)	2 (0.8%)

Cholecystitis severity					
Grade 1	152 (51.9%)	399 (54.2%)	12 (19.7%)	3 (30.0%)	134 (54.0%)
Grade 2	126 (43.0%)	316 (42.9%)	35 (42.9%)	5 (50.0%)	109 (44.0%)
Grade 3	4 (1.4%)	6 (0.8%)	11 (18.0%)	2 (20.0%)	3 (1.2%)
Data missing	11 (3.8%)	15 (2.0%)	3 (4.9%)	0 (0%)	2 (0.8%)

Median hospital stay	4	4	9	8.5	3

Complications related to cholecystostomy and/or cholecystectomy (Clavien-Dindo)					
2		51 (6.9%)	2 (3.3%)	0 (0%)	10 (4.0%)
3		70 (9.5%)	1 (1.6%)	2 (20.0%)^*∗*^	24 (9.7%)
4		3 (0.4%)	0 (0%)	0 (0%)	2 (0.8%)
5		2 (0.3%)	1 (1.6%)	0 (0%)	0 (0%)

^*∗*^Both registered complications related to the cholecystectomy.

**(a):** 

	Cholecystostomy group (*N* = 71)	Acute cholecystectomy group (*N* = 736)	Difference	*P* (univariate analysis)	*P* (multivariate analysis)
Mean hospital stay, days (95% confidence interval)	11.4 (8.9–13.8)	5.1 (4.8–5.4)	6.3 (3.6–9.0)	<0.001	<0.001

**(b):** 

	Cholecystostomy group (*N* = 71)	Acute cholecystectomy group (*N* = 736)	Odds ratio (univariate analysis)	Odds ratio (multivariate analysis)
Complication rate (Clavien-Dindo ≥2)	2 (2.8%)	126 (17.1%)	7.1 (1.7–29.4)	15.1 (3.4–66.8)
